# Comparative mRNA Expression of eEF1A Isoforms and a PI3K/Akt/mTOR Pathway in a Cellular Model of Parkinson's Disease

**DOI:** 10.1155/2016/8716016

**Published:** 2016-02-14

**Authors:** Kawinthra Khwanraj, Suriyat Madlah, Khwanthana Grataitong, Permphan Dharmasaroja

**Affiliations:** Department of Anatomy, Faculty of Science, Mahidol University, Bangkok 10400, Thailand

## Abstract

The PI3K/Akt/mTOR pathway is one of dysregulated pathways in Parkinson's disease (PD). Previous studies in nonneuronal cells showed that Akt regulation can be increased by eukaryotic protein elongation factor 1 alpha 2 (eEF1A2). eEF1A2 is proposed to contribute protection against apoptotic death, likely through activation of the PI3K/Akt pathway. Whether eEF1A2 plays a role in the prevention of cell death in PD has not been investigated. Recently, gene profiling on dopaminergic neurons from postmortem PD patients showed both upregulation and downregulation of some PI3K and mTOR genes. In this paper, the expression of all gene members of the PI3K/Akt/mTOR pathway in relation to those of the eEF1A isoforms in a cellular model of PD was investigated at the mRNA level. The results showed a similar trend of upregulation of genes of the eEF1A isoforms (*eEF1A1 *and* eEF1A2*) and of the PI3K (classes I–III)/Akt (*Akt1*,* Akt2*, and* Akt3*)/mTOR (*mTORC1 *and* mTORC2*) pathway in both nondifferentiated and differentiated SH-SY5Y dopaminergic cells treated with 1-methyl-4-phenylpyridinium (MPP^+^). Upregulation of* eEF1A2*,* Akt1*, and* mTORC1 *was consistent with the relative increase of eEF1A2, Akt, phospho-Akt, and mTORC1 proteins. The possible role of eEF1A isoforms in the regulation of the PI3K/Akt/mTOR pathway in PD is discussed.

## 1. Introduction

The phosphatidylinositol 3-kinase (PI3K)/Akt serine/threonine protein kinase (Akt)/mammalian target of rapamycin kinase (mTOR) pathway is a crucial signaling mediator, which seems to serve as a connection between aspects of neuronal proliferation, differentiation, and programmed apoptotic death [1, 2]. The PI3K/Akt/mTOR system is also viewed as one amongst the key neuroprotective signaling pathways [3]. Activation of the PI3K/Akt pathway, as a neuroprotective effect of caffeine, has been shown in a cellular model of Parkinson's disease (PD) [4]. Treatment with 1-methyl-4-phenylpyridinium (MPP^+^) leads to increased dephosphorylation of mTOR and its substrate, p70S6K, in both control and G2019S LRRK2-mutant skin fibroblasts [5]. Several studies also support possible pharmacological prevention of dopaminergic neuron death through Akt and mTOR [6–9]. Inhibition of mTOR using rapamycin has been reported to suppress PINK1 and Parkin pathology [10]. After activation, mTOR further activates its downstream effectors that regulate the transcription of many genes and the synthesis of proteins [11].

eEF1A (eukaryotic protein elongation factor 1 alpha) proteins are GTP-binding proteins that bind to amino-acylated tRNA and deliver them to the ribosome during the elongation process of protein translation. There are two isoforms of eEF1A: eEF1A1 and eEF1A2, which share >90% sequence identity [12]. eEF1A1 is widely expressed, whereas eEF1A2 is specifically expressed in the brain, heart, and skeletal muscles [12–14]. In addition to their canonical function in translation elongation, binding of eEF1A1 to actin filaments and microtubules suggests its role in actin binding/bundling and microtubule bundling/severing [15]. eEF1A2 has been reported to interact with M4 muscarinic acetylcholine receptors (mAChR) in the brain [16]. eEF1A2 is considered an important human oncogene. Its increased expression has been found in tumors of the ovary, breast, and lung [17–20]. The molecular mechanisms underlying progressive dopaminergic cell death in the nigrostriatal pathway of PD patients are not completely understood. Recent genetic and functional analyses show that there are pathogenic overlaps between cancer and PD [21]. In the BT549 human breast cancer cell line, eEF1A2 regulates cancer formation through Akt and PI3K-dependent remodeling of the cytoskeleton [22]. In mouse plasmacytomas,* Eef1a2* regulates tumor growth and proliferation through activation of the JAK/STAT and Akt signaling pathways [23].

Overexpression of eEF1A2 also protects cells against apoptotic death induced by oxidative stress [24]. eEF1A2 is found to interact with peroxiredoxin-I (Prdx-I), a typical 2-Cys peroxiredoxin, widely expressed in all mammalian cells [25]. Prdx-I functionally protects cells from oxidative stress-induced cell damage by reducing a range of reactive oxygen species (ROS) production. The interaction of eEF1A2 with Prdx-I provides an increase in protection from oxidative stress-induced death. This protection is correlated with the decrease in cleavage of caspases 3 and 8 and elevated expression of prosurvival Akt.

Whether eEF1A1 or eEF1A2 plays a role in the prevention of cell death in PD has not been investigated. The aim of our study was to analyze the pattern of gene expression for eEF1A isoforms in relation to that of the PI3K/Akt/mTOR pathway. Thus, we evaluated real-time mRNA expression of PI3K isoforms: PI3K classes I, II, and III; Akt isoforms: Akt1 and Akt2; and mTOR isoforms: mTORC1 and mTORC2, together with the expression of eEF1A1 and eEF1A2 mRNAs, using an MPP^+^-induced SH-SY5Y cellular model of PD. We expected that there would be a similar trend of increased expression of eEF1A isoforms and of the PI3K/Akt/mTOR pathway. Indirectly we expected the result to suggest whether any eEF1A isoforms could become a novel prosurvival factor in protecting dopaminergic cells against toxin-induced neuronal death.

## 2. Materials and Methods

### 2.1. Materials

Eagle's Minimum Essential Medium (MEM), Nutrient Mixture Ham's F12 medium (F12), fetal bovine serum (FBS), and other supplements for cell culture were purchased from Gibco (Gaithersburg, MD, USA). Retinoic acid (RA), 1-methyl-4-phenylpyridinium (MPP^+^), 3-(4,5-dimethylthiazol-2-yl)-2,5-diphenyltetrazolium bromide (MTT), and DMSO were purchased from Sigma-Aldrich (St. Louis, MO, USA). Primers for real-time RT-PCR were synthesized by and purchased from Biolegio (Nijmegen, Netherlands). Antibodies against Akt, phosphorylated Akt, phosphorylated mTORC1, and tyrosine hydroxylase (TH) were purchased from Cell Signaling Technology (Danvers, MA, USA). Antibody against eEF1A2 and anti-*β*-actin were purchased from Sigma-Aldrich (St. Louis, MO, USA). Horseradish peroxidase- (HRP-) conjugated anti-rabbit IgG and HRP-conjugated anti-mouse IgG were purchased from Abcam (Cambridge, UK) and Invitrogen (Eugene, OR, USA), respectively.

### 2.2. Culture and Treatment of SH-SY5Y Cells

SH-SY5Y human neuroblastoma cells were grown in 1 : 1 mixture of MEM and F12, supplemented with 10% heat-activated FBS. Cells were separated into two groups: nondifferentiated and differentiated cells. For neuronal differentiation of SH-SY5Y cells, RA was added a day after plating at final concentration 10 *μ*M in MEM-F12 with 10% FBS and maintained for three days. Neuronal differentiation was demonstrated as an increase in tyrosine hydroxylase (TH) expression using immunostaining. Thereafter, nondifferentiated and differentiated SH-SY5Y cells were exposed to 125, 250, 500, 1000, and 2000 *μ*M of MPP^+^ for 24 hours. The control groups for nondifferentiated and differentiated SH-SY5Y cells were treated with the same medium without MPP^+^.

### 2.3. Immunostaining for Tyrosine Hydroxylase

SH-SY5Y cells were fixed in 4% paraformaldehyde for 15 minutes at room temperature followed by sequential incubation with permeabilizing solution (0.2% Triton X-100) in PBS for 30 minutes at room temperature. Then, cultures were washed again with PBS and incubated in blocking solution (3% BSA in 0.5% Tween 20 in PBS) for 30 minutes. Cells were incubated with rabbit polyclonal antibody against TH (1 : 200 dilution in blocking solution; Merck Millipore AB152) overnight at 4°C. After washing, cells were incubated with 1 : 500 dilution of Alexa 488-conjugate secondary antibody for 1 hour at room temperature. Coverslips were then mounted with Vectashield antifading mounting medium with DAPI (Vector laboratories, CA). Cells were visualized under a confocal laser-scanning microscope (Olympus model FV 1000; Tokyo, Japan).

### 2.4. Cell Viability Assay

SH-SY5Y cells were seeded onto a 96-well plate at a density of 1 × 10^4^ cells/well in 200 *μ*L of medium and incubated at 37°C under 5% CO_2_. After exposure to MPP^+^, cell viability was measured by MTT colorimetric assay. This method was based on the reduction of tetra ring of MTT by mitochondrial dehydrogenases with NADH in the active mitochondria, yielding a blue formazan product, which can be measured spectrophotometrically. After incubation, 20 *μ*L of MTT (5 mg/mL) was added to each well and the cells were cultured for another 4 h; then medium was removed and 100 *μ*L of DMSO was added to each well to dissolve the formazan. The color reaction was measured at wavelength 570 nm with a reference at 690 nm using the VERSAmax Tunable microplate reader with SoftMax Pro software (Molecular Devices, Sunnyvale, CA, USA).

### 2.5. Real-Time Quantitative PCR Analysis

SH-SY5Y cells were seeded onto 6-well plates. Total mRNA was extracted from the pellet using PARIS kit according to the supplier's instructions. DNase I treatment was performed after RNA extraction, followed by heat inactivation. The quantity and purity of RNA were determined by optical density measurements at OD A260/A280 ratio with 1.8 or above using Nanodrop 2000 spectrophotometer (Thermo Fisher Scientific Inc., Wilmington, DE, USA). Later, the cDNA was synthesized from 1 *μ*g of RNA using Masterscript RT-PCR System (5 PRIME, Gaithersburg, MD, USA), according to the manufacturer's instructions, and stored at −20°C until assay. KAPA SYBR FAST qPCR kit (Kapa Biosystems, Woburn, MA, USA) was used for real-time PCR quantification. The 20 *μ*L real-time PCR reaction mixture contained 20 ng cDNA template, 10 *μ*L of 1x KAPA SYBR FAST qPCR master mix, 200 nM of forward and reverse primers, and PCR-grade water. *β*-Actin was used as a reference gene. The sequences of the primers are shown in Table 1 [26–28]. The reaction was performed in the Applied Biosystems 7500 real-time PCR system (Applied Biosystems, Foster City, CA, USA) with the PCR cycling conditions as follows: 3 minutes for enzyme activation at 95°C, 40 cycles of 3 seconds for initial denaturation at 95°C, and annealing/extension at 60°C for 1 minute. Melting curve analysis was performed to verify specificity of each primer after PCR to ensure amplification specificity. The threshold cycle (Ct) number was determined and used in the comparative Ct method. The relative quantity of the target gene was estimated by the 2^−ΔΔCt^ method. All data was analyzed by the ABI 7500 software, version 2.0.

### 2.6. Western Blot Analysis

SH-SY5Y cells were seeded at an initial concentration of 2 × 10^5^ cells/well in 6-well plates. After treatment with MPP^+^ for 24 hours, cells were trypsinized and centrifuged to collect the pellet. Cold RIPA buffer (50 mM Tris PH 7.4, 150 mM NaCl, 1% triton X-100, 0.1% SDS, 1% sodium deoxycholate, 5 mM EDTA, 30 mM Na_2_HPO_4_, and 50 mM NaF) was added to the pellets, mixed, and incubated on ice for 20 minutes, followed by centrifugation. The supernatants were collected and kept at −80°C until being used. Sixty *μ*g of protein extracts was separated in 8% sodium dodecyl sulfate-polyacrylamide gel electrophoresis (SDS-PAGE) and then transferred onto a nitrocellulose membrane. The membrane was blocked with 5% nonfat dry milk in 1x Tris-buffered saline containing 0.1% Tween 20 for 2 hours. It was then incubated with rabbit polyclonal anti-EEF1A2 (1 : 2000 dilution), rabbit monoclonal anti-Akt (1 : 1000 dilution), rabbit monoclonal anti-phosphorylated Akt at serine 473 (1 : 1000 dilution), rabbit monoclonal anti-phosphorylated mTORC1 at Ser 2448 (1 : 1000 dilution), or mouse monoclonal anti-*β*-actin (1 : 5000 dilution) overnight at 4°C. Subsequently the membrane was incubated with HRP-conjugated secondary antibodies (1 : 2000–5000 dilution) for 2 hours at 4°C. The bands were visualized using ECL Prime Western Blotting Detection (GE Healthcare, Buckinghamshire, UK). The signal intensities were determined by densitometry using Image-J software (National Institutes of Health, Bethesda, Maryland, USA).

### 2.7. Statistical Analysis

Experiments on cell viability assay and real-time quantitative PCR were repeated three times in triplicate measurement. Statistical analyses were performed with one-way ANOVA test followed by a post hoc analysis (Tukey's multiple comparison test) using GraphPad Prism 5 Software for Windows (GraphPad Software, Inc., San Diego, CA, USA). All values were expressed as mean ± standard error of the mean (mean ± SEM) for each group. A *P* less than 0.05 was considered statistically significant.

## 3. Results

### 3.1. Effect of MPP^+^ on SH-SY5Y Cell Viability

**Figure 1:**
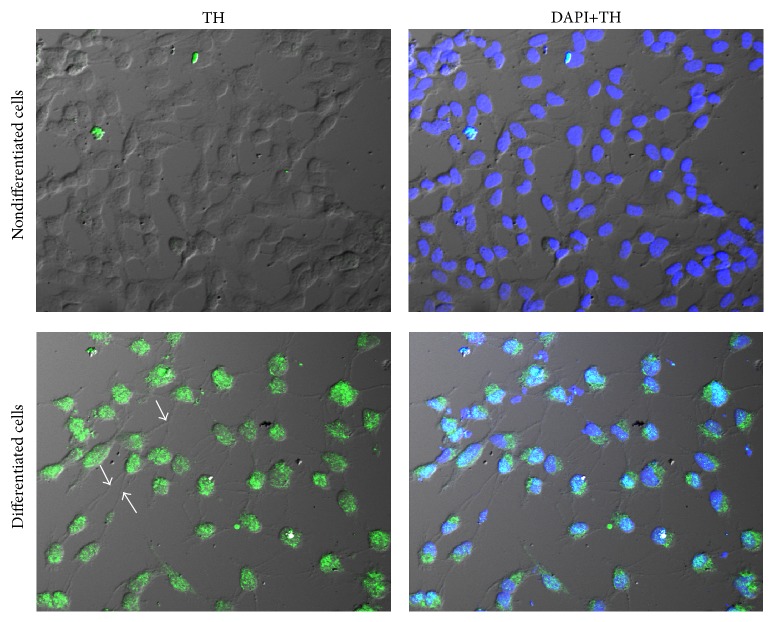
Expression of tyrosine hydroxylase in undifferentiated and differentiated SH-SY5Y cells. Cells were differentiated in 10 *μ*M retinoic acid (RA). After differentiation for 3 days, expression of tyrosine hydroxylase (TH) was visualized through immunostaining in undifferentiated cells and RA-differentiated cells. Nuclei were stained using DAPI. White arrows indicate areas of neurite outgrowth.

**Figure 2:**
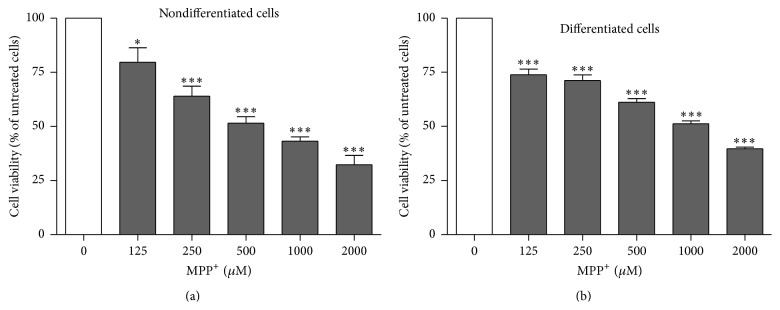
Effect of MPP^+^ on cell viability of nondifferentiated SH-SY5Y cells (a) and effect of MPP^+^ on cell viability of differentiated SH-SY5Y cells (b). Cells were exposed to 125, 250, 500, 1000, and 2000 *μ*M of MPP^+^ for 24 hours and cell viability was measured with an MTT assay. For neuronal differentiation, SH-SY5Y cells were treated with 10 *μ*M retinoic acid for three days before exposure to MPP^+^. The control groups for nondifferentiated and differentiated SH-SY5Y cells were treated with the same medium without MPP^+^. MTT assays were repeated three times in triplicate measurement. Data are shown as mean ± SEM. *∗* indicates *P* < 0.05 and *∗∗∗* indicates *P* < 0.001 versus 0 *μ*m of MPP^+^.

To investigate the influence of MPP^+^ on cell viability in nondifferentiated and differentiated SH-SY5Y neuroblastoma cells, cells were exposed to 125, 250, 500, 1000, and 2000 *μ*M MPP^+^ for 24 hours. For differentiated cells, the cells were incubated with 10 *μ*M RA for 3 days to induce neuronal differentiation prior to exposure to various dosages of MPP^+^. Differentiation of SH-SY5Y cells to a neuronal phenotype was confirmed by an increase in the expression of tyrosine hydroxylase and neuritic outgrowth (Figure 1). Cell viability was examined with an MTT assay. Cell viability was significantly reduced by all dosages of MPP^+^ in both nondifferentiated and differentiated cells (Figure 2). In nondifferentiated cells, exposure to 500 *μ*M MPP^+^ led to reduction of viability by approximately 50%. However, 1000 *μ*M MPP^+^ was required to reduce the viability of differentiated cells to about 50%. Overall, higher dosages of MPP^+^ were required to induce cell death in differentiated cells compared with nondifferentiated cells. Thus, 500 and 1000 *μ*M MPP^+^ were used to treat nondifferentiated and differentiated cells, respectively, in later experiments.

### 3.2. Expression of eEF1A1 and eEF1A2

**Figure 3:**
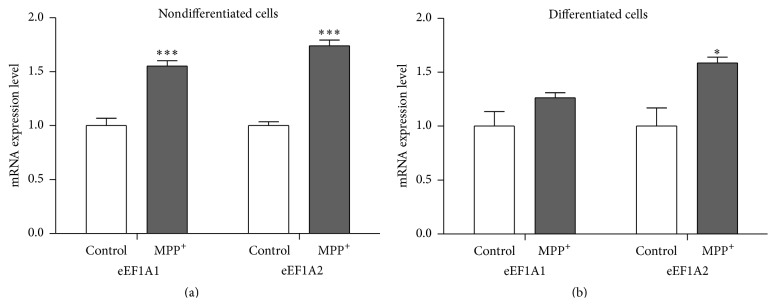
mRNA expression levels of* eEF1A1* and* eEF1A2* in nondifferentiated SH-SY5Y cells (a) and mRNA expression levels of* eEF1A1* and* eEF1A2* in differentiated SH-SY5Y cells (b). For neuronal differentiation, SH-SY5Y cells were treated with 10 *μ*M retinoic acid for three days before exposure to MPP^+^. The control groups for nondifferentiated and differentiated SH-SY5Y cells were treated with the same medium without MPP^+^. Real-time quantitative PCRs were repeated three times in triplicate measurement. Data are expressed as relative to a control and shown as mean ± SEM. *∗* indicates *P* < 0.05 and *∗∗∗* indicates *P* < 0.001 versus control of each group.

**Figure 4:**
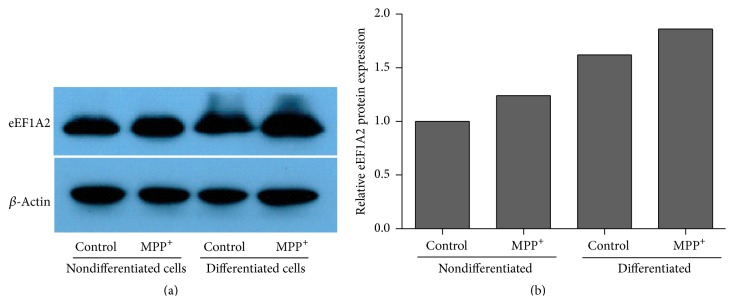
Relative protein expression of eEF1A2 in nondifferentiated and differentiated SH-SY5Y cells by Western blot (a). For neuronal differentiation, SH-SY5Y cells were treated with 10 *μ*M retinoic acid for three days before exposure to MPP^+^. The control groups for nondifferentiated and differentiated SH-SY5Y cells were treated with the same medium without MPP^+^. (b) represents the quantification of the band density normalized to actin, relative to a nondifferentiated control.

Quantitative real-time PCR showed that eEF1A1 and eEF1A2 mRNAs were significantly increased in nondifferentiated SH-SY5Y cells after 24-hour exposure to MPP^+^ (*P* < 0.001; Figure 3(a)). In differentiated cells, a significant increase was observed only for eEF1A2 mRNA (*P* < 0.05; Figure 3(b)). As the eEF1A2 isoform has been reported to be involved in protection from oxidative stress-induced death, Western blotting was performed subsequently to evaluate eEF1A2 expression at the protein level. The result showed relatively increased levels of the eEF1A2 protein in both nondifferentiated and differentiated cells treated with MPP^+^ (Figure 4), which is consistent with the mRNA result.

### 3.3. Expression of Akt1, Akt2, and Akt3

**Figure 5:**
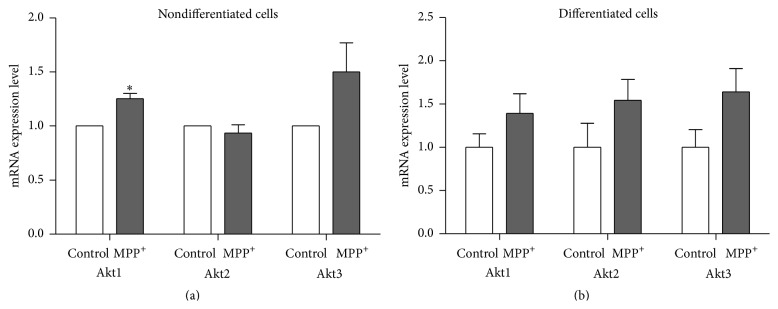
mRNA expression levels of* Akt1*,* Akt2*, and* Akt3* in nondifferentiated SH-SY5Y cells (a) and mRNA expression levels of* Akt1*,* Akt2*, and* Akt3* in differentiated SH-SY5Y cells (b). For neuronal differentiation, SH-SY5Y cells were treated with 10 *μ*M retinoic acid for three days before exposure to MPP^+^. The control groups for nondifferentiated and differentiated SH-SY5Y cells were treated with the same medium without MPP^+^. Real-time quantitative PCRs were repeated three times in triplicate measurement. Data are expressed as relative to a control and shown as mean ± SEM. *∗* indicates *P* < 0.05 versus control of each group.

**Figure 6:**
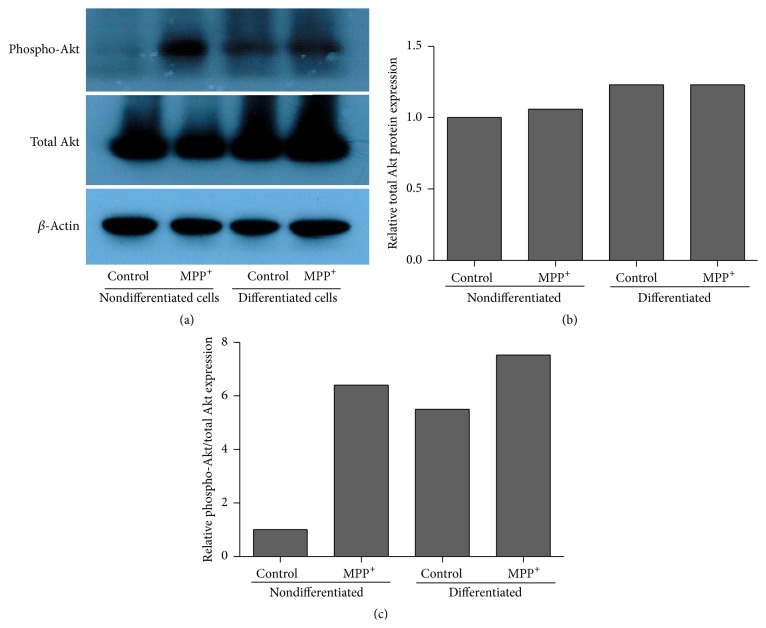
Relative protein expression of total Akt and phospho-Akt in nondifferentiated and differentiated SH-SY5Y cells by Western blot. For neuronal differentiation, SH-SY5Y cells were treated with 10 *μ*M retinoic acid for three days before exposure to MPP^+^. The control groups for nondifferentiated and differentiated SH-SY5Y cells were treated with the same medium without MPP^+^. (b) and (c) represent the quantification of the band density of total Akt and phospho-Akt, respectively. The band density was normalized to actin and relative to a nondifferentiated control.

After 24-hour exposure to MPP^+^, a tendency of increased expression of Akt1, Akt2, and Akt3 mRNA was observed in differentiated SH-SY5Y cells, although this could not be confirmed statistically (Figure 5(b)). In nondifferentiated cells, significantly increased expression was observed for Akt1 mRNA (*P* < 0.05) and relatively increased expression was observed for Akt3 mRNA (Figure 5(a)). Western blotting using antibodies against Akt did not show significantly increased levels of total Akt protein in both nondifferentiated and differentiated cells treated with MPP^+^ (Figure 6). However, when using an antibody against phospho-Akt1 (Ser-473), which can also bind to equivalent residues of Akt2 and Akt3, the result showed relatively increased levels of phospho-Akt/total Akt protein in both nondifferentiated and differentiated cells treated with MPP^+^.

### 3.4. Expression of mTORC1 and mTORC2

**Figure 7:**
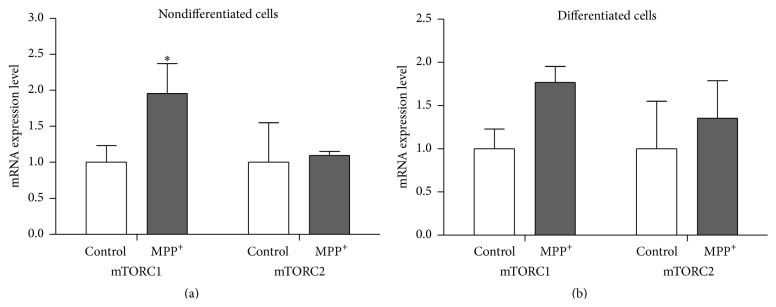
mRNA expression levels of* mTORC1* and* mTORC2* in nondifferentiated SH-SY5Y cells (a) and mRNA expression levels of* mTORC1* and* mTORC2* in differentiated SH-SY5Y cells (b). For neuronal differentiation, SH-SY5Y cells were treated with 10 *μ*M retinoic acid for three days before exposure to MPP^+^. The control groups for nondifferentiated and differentiated SH-SY5Y cells were treated with the same medium without MPP^+^. Real-time quantitative PCRs were repeated three times in triplicate measurement. Data are expressed as relative to a control and shown as mean ± SEM. *∗* indicates *P* < 0.05 versus control of each group.

**Figure 8:**
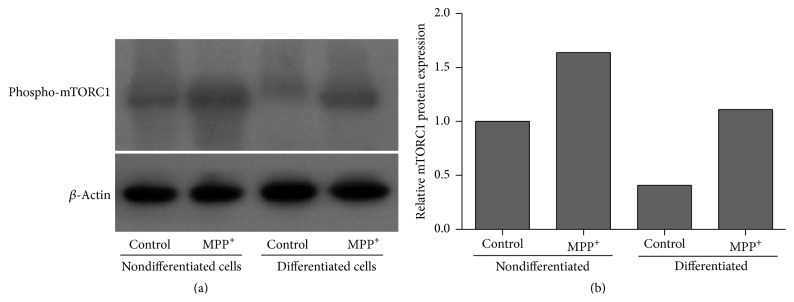
Relative protein expression of phospho-mTORC1 in nondifferentiated and differentiated SH-SY5Y cells by Western blot. For neuronal differentiation, SH-SY5Y cells were treated with 10 *μ*M retinoic acid for three days before exposure to MPP^+^. The control groups for nondifferentiated and differentiated SH-SY5Y cells were treated with the same medium without MPP^+^. (b) represents the quantification of the band density normalized to actin, relative to a nondifferentiated control.

Relatively increased mRNA expression levels of mTORC1 and mTORC2 were observed in both nondifferentiated and differentiated SH-SY5Y cells after exposure to MPP^+^ for 24 hours, compared to untreated cells (Figure 7). Significantly increased expression of mTORC1 mRNA was seen in nondifferentiated cells (*P* < 0.05; Figure 6(a)). Western blotting using an antibody against Ser-2448 residue of phospho-mTORC1 also showed relatively increased levels of phospho-mTORC1 in both nondifferentiated and differentiated cells (Figure 8), which is consistent with the mTORC1 mRNA result.

### 3.5. mRNA Expression of PI3Ks

**Figure 9:**
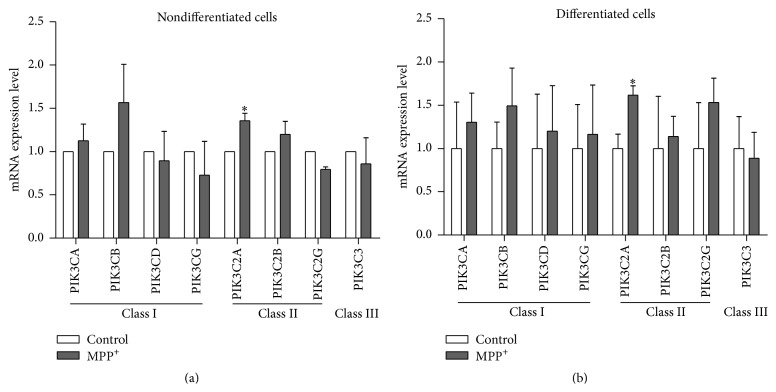
mRNA expression levels of genes of PI3K classes I, II, and III in nondifferentiated SH-SY5Y cells (a) and mRNA expression levels of genes of PI3K classes I, II, and III in differentiated SH-SY5Y cells (b). For neuronal differentiation, SH-SY5Y cells were treated with 10 *μ*M retinoic acid for three days before exposure to MPP^+^. The control groups for nondifferentiated and differentiated SH-SY5Y cells were treated with the same medium without MPP^+^. Real-time quantitative PCRs were repeated three times in triplicate measurement. Data are expressed as relative to a control and shown as mean ± SEM. *∗* indicates *P* < 0.05 versus control of each group.

All genes encoding the members of the three human PI3K classes were studied. Class I included* PIK3CA*,* PIK3CB*,* PIK3CD*, and* PIK3CG* genes. Class II included* PIK3C2A*,* PIK3C2B*, and* PIK3C2G*. Class III included* PIK3C3*. Quantitative real-time PCR showed that all of the genes encoding class I and II PI3Ks were relatively increased in differentiated SH-SY5Y cells treated with MPP^+^ for 24 hours when compared to untreated cells (Figure 9(b)). Expression of* PIK3C* was relatively decreased in both differentiated and nondifferentiated cells (Figure 9). Expression of class I and II PI3K genes was somewhat varied in nondifferentiated cells (Figure 9(a)). It should be noted that expression of* PIK3C2A* was significantly increased in both nondifferentiated and differentiated cells treated with MPP^+^ (*P* < 0.05).

## 4. Discussion

As expected, we observed similar mRNA expression between both eEF1A isoforms and PI3K/Akt/mTOR in both nondifferentiated and differentiated SH-SY5Y cells. Differentiated and nondifferentiated SH-SY5Y cells have been widely used as a cell model of dopaminergic neurons for PD research. A previous study suggested that differentiated SH-SY5Y cells, induced by low serum and RA treatment, represent a more suitable experimental model for studying the molecular and cellular mechanisms underlying the pathophysiology of PD, as shown by increasing sensitivity to 6-hydroxydopamine (6-OHDA) toxicity during the differentiation process [29]. In contrast, another study suggested that nondifferentiated SH-SY5Y is more appropriate [30]. In the latter study, nondifferentiated SH-SY5Y cells were susceptible to 6-OHDA and MPP^+^, while RA-differentiated SH-SY5Y cells conferred higher tolerance, potentially by upregulating survival signaling, including the Akt pathway. Thus, we used both nondifferentiated and differentiated SH-SY5Y cells for comparison in the present study.

An interesting finding of this study is the significantly upregulated mRNA expression of eEF1A2 isoform of eEF1A in SH-SY5Y cells after exposure to MPP^+^. The upregulation was observed in both nondifferentiated and differentiated cells. In contrast, significant upregulation of eEF1A1 was observed in nondifferentiated cells, but not in differentiated cells. The explanation for eEF1A1 upregulation in nondifferentiated cells after MPP^+^ exposure remains to be investigated. Nondifferentiated SH-SY5Y cells contain characteristics of cancer. Upregulation of eEF1A1 may reflect a cellular response to increased synthesis of several intracellular proteins involving pro- and antisurvival processes. The same explanation may be applied to the upregulation of eEF1A2 in nondifferentiated cells. Another hypothesis for eEF1A2 upregulations observed in differentiated cells after MPP^+^ exposure is its role in the protection of cells against oxidative stress. The differentiated SH-SY5Y cells acquire neuronal phenotypes after treatment with RA [29, 30]. RA treatment confers SH-SY5Y cells with higher tolerance to MPP^+^, potentially by upregulating the survival Akt signaling pathway. Significant upregulation of eEF1A2 but not eEF1A1 in MPP^+^-treated differentiated SH-SY5Y cells, whose phenotype is dopaminergic, may reflect differential functions of these eEF1A isoforms.

eEF1A2 has been shown to activate prosurvival Akt in a PI3K-dependent manner in a breast cancer cell line [22]. eEF1A2 has also been shown to involve a reduction of apoptosis in mouse plasmacytoma cell lines via activation of Akt [23]. The role of eEF1A2 in resistance to apoptosis has been supported in other cell types [25, 31]. Recently, expression profiling and pathway study using microarray analysis of dopaminergic neurons removed from the frozen brains of patients with PD has shown that there are several significantly dysregulated pathways. Among these pathways, the PI3K/Akt/mTOR signaling pathway is considered a central hub. To associate the prosurvival role of eEF1A2 with that of the PI3K/Akt/mTOR pathway, we investigated the relative expression of isoforms further for PI3K, Akt, and mTOR families at the mRNA level.

In the present study, we found that all of the three Akt isoforms were relatively increased in their expression in differentiated SH-SY5Y cells after exposure to MPP^+^. Although the isoforms share a high degree of structural homology, several studies suggested distinct roles for each of the Akt family kinases [32–35]. It is recognized that Akt1 is involved in the prosurvival processes of the cell, including antiapoptosis. The role of Akt2 and Akt3 in the induction of caspase-dependent apoptosis has been reported [36, 37]. Studies of the role of eEF1A2 on activation of Akt are mainly focused on the Akt1 isoform, whose phosphorylation sites are at Thr 308 and Ser 473 [22, 23]. Western blotting using the antibody against phospho-Akt (Ser 473) in our study also showed an increased expression of phospho-Akt protein in MPP^+^-treated SH-SY5Y cells, those being both nondifferentiated and differentiated, similar to previous studies [4, 38]. It should be noted that antibody against the Ser-473 residue of Akt1 might bind to equivalent residues of Akt2 and Akt3. There is evidence of differential regulation of the isoforms after induction using a common Akt activator [39]. Whether eEF1A2 is a common Akt activator and possibly contributes to isoform specific regulation of Akt in PD and other cellular processes remains unknown at this time.

We investigated the downstream signaling of Akt further. Activation of the PI3K/Akt/pathway promotes survival and neuronal protection by mTOR activation [40]. mTOR, the mammalian target of rapamycin, exists in two distinct complexes, mTORC1 and mTORC2 [41]. The PI3K/Akt pathway is a major upstream modulator of mTORC1 [42]. mTORC1 is the key player in the modulation of the autophagic process, with mTORC2 implicated in Akt activation [40]. Studies on neuroblastoma cellular models of PD showed the reduced phosphorylation of the major autophagy repressor mTORC1 after exposure to either 2–12 hours of 1000 *μ*M MPP^+^ for differentiated cells or 8–16 hours of 50 *μ*M 6-OHDA for nondifferentiated cells [43, 44]. Less is known about the role of mTORC2 in PD. Our Western blotting using the antibody against phospho-mTORC1 also showed an increased expression of the phospho-mTORC1 protein in SH-SY5Y cells, after 24-hour exposure to 500 *μ*M MPP^+^ and 1000 *μ*M MPP^+^ for nondifferentiated and differentiated cells, respectively. The protein results were consistent with the mRNA results. Discrepancies between our and previous studies remain to be investigated. There has not been any study of the relationship between eEF1A isoforms and mTOR activation in PD. Evidence from acute brain slices has shown that the induction of protein synthesis-dependent hippocampal long-term potentiation that increases the local expression of eEF1A1 is mediated by the mTORC1 signaling [45]. A study of pheochromocytoma cells also showed that stimulation with a nerve growth factor causes preferential synthesis of eEF1A1 over eEF1A2. This process is mediated by mTORC1 [46]. Our results provide additional information, at the mRNA level, regarding the correlation between the expression of isoforms of eEF1A and mTOR in PD.

Next, we investigated the PI3Ks, the upstream regulator for Akt activation. There are three classes (I–III) of PI3Ks in mammals. Involvement of PI3Ks in various cellular models of PD is usually investigated using a PI3K inhibitor, LY294002, which inhibits most of members of the PI3K classes [46]. Data is lacking with regard to the predominant class of PI3Ks involved in neuroprotection. Our present data showed, at the mRNA level, that almost all members of PI3Ks, except class III, were likely to be upregulated when SH-SY5Y cells were exposed to MPP^+^.

Parkinson's disease is a complex disorder. Data from microarray-based gene expression profiling on human substantia nigra dopaminergic neurons dissected using laser microdissection showed both upregulation and downregulation of genes of the PI3K/Akt/mTOR signaling pathway in the neurons of PD patients [6, 47]. For example,* PIK3C2G* and* PIK3R2*, members of class II and I PI3Ks, respectively, are upregulated in human dopaminergic neurons [47]. We observed considerable variability of mRNA expression levels across the sample populations. The results derived from within each sample, represented as mean ± SD, were statistically significant (data not shown), while those derived from analysis across samples, represented as mean ± SEM, were mostly insignificant. However, there was overall consistency among each gene studied. This observation was similar to the results of a previous study performed using TaqMan real-time PCR to validate the microarray data [47]. It should be also emphasized that mRNA expression levels reveal information at the level of transcriptional activation of genes but do not provide much about actual protein levels or functions. Additional work on the protein expression for each isoform of eEF1A and members of the PI3K/Akt/mTOR pathway is necessary to confirm the involvement of these genes and proteins in the mechanistic network of PD.

## 5. Conclusions

Our mRNA expression analysis is the first study grounded on the expression of gene members of the PI3K/Akt/mTOR signaling pathway in relation to those of the eEF1A isoforms in a toxin-induced cellular model of PD. Both eEF1A1 and eEF1A2 may be involved in the response to MPP^+^ of dopaminergic cells, although eEF1A2 seems to be predominant. Association between eEF1A2 and PI3K/Akt/mTOR has been supported in other cell types [22, 48]. Whether or not eEF1A2 could be a potential candidate for pharmacological intervention remains to be seen. Further studies to address the relationship or interaction between eEF1A isoforms and PI3K/Akt/mTOR members, including other cell survival pathways, should be conducted in available cellular or animal models of PD. In addition, overexpression study for eEF1A may accentuate the effects of the PI3K/Akt/mTOR pathway. The current results could be a useful adjunct to support previous studies using the microarray-based gene profiling analysis, although validation of the gene expression in human dopaminergic neurons of PD patients remains to be performed. Lastly, our study on the PI3K/Akt/mTOR genes in SH-SY5Y dopaminergic cells also provides a reference gene framework for researchers working in PD. The expression pattern will offer useful clues about the function of the PI3K/Akt/mTOR pathway during PD pathogenesis and regulation of neuroprotection.

## Figures and Tables

**Table 1:** List of primer pairs used for real-time quantitative PCR.

Gene symbol	Primers sequences 5′-3′
*eEF1A1*	GGCATACCCGAGAGCATG
	AGGCATGTTAGCACTTGGC

*eEF1A2*	GAGCCCTCCCCCAACATGCC
	ATGTTCACTGGCGCAAAGGTCAC

*PIK3CA*	TGGATGCTCTACAGGGCTTT
	GTCTGGGTTCTCCCAATTCA

*PIK3CB*	GCATTAAAAGGGAGCGAGTG
	CATGCCGTCGTAAAATCAGA

*PIK3CD*	CTGGCTGAAGTCCAAGAACC
	CTCGGATCATGATGTTGTCG

*PIK3CG*	ATACCATGATAGCGCCCTTG
	AATCACAGCGAACCTCTGCT

*PIK3C2A*	GAAAAACGAGGAATCCGACA
	CAGGGTTACTCCACCCAAGA

*PIK3C2B*	TTCCCTAGTCGCTTCGTGAT
	CAGTGGGTGGAAGAAGGTGT

*PIK3C2G*	TTCATCTCCCAGATGGCTCT
	AGTGGGGTCCGTACATTTTG

*PIK3C3*	TCAGCCAAGCATTGTTGAAG
	TCCACTTTCGCGTTGTACTG

*Akt1*	TCTATGGCGCTGAGATTGTG
	CTTAATGTGCCCGTCCTTGT

*Akt2*	TATACCGCGACATCAAGCTG
	GGTCCCACAGAAGGTTTTCA

*Akt3*	TGGACAAAGATGGCCACATA
	ACTGCTCGGCCATAGTCATT

*mTORC1*	AGGCCGCATTGTCTCTATCAA
	GCAGTAAATGCAGGTAGTCATCCA

*mTORC2*	TCTACCACGACAGCCCGGCA
	TGGGGGCCCCGTTCCATCAT

*β-Actin*	CATGTACGTTGCTATCCAGGC
	CTCCTTAATGTCACGCACGAT
